# IL-21 production by CD4^+^ effector T cells and frequency of circulating follicular helper T cells are increased in type 1 diabetes patients

**DOI:** 10.1007/s00125-015-3509-8

**Published:** 2015-02-06

**Authors:** Ricardo C. Ferreira, Henry Z. Simons, Whitney S. Thompson, Antony J. Cutler, Xaquin Castro Dopico, Deborah J. Smyth, Meghavi Mashar, Helen Schuilenburg, Neil M. Walker, David B. Dunger, Chris Wallace, John A. Todd, Linda S. Wicker, Marcin L. Pekalski

**Affiliations:** 1 JDRF/Wellcome Trust Diabetes and Inflammation Laboratory, Department of Medical Genetics, NIHR Cambridge Biomedical Research Centre, Cambridge Institute for Medical Research, University of Cambridge, WT/MRC Building, Addenbrooke’s Hospital, Hills Road, Cambridge, CB2 0XY UK; 2 Department of Paediatrics, School of Clinical Medicine, University of Cambridge, Cambridge, UK

**Keywords:** Human, IFN-γ, IL-17, IL-21, Immunophenotyping, T follicular helper cell, Type 1 diabetes

## Abstract

**Aims/hypothesis:**

Type 1 diabetes results from the autoimmune destruction of insulin-secreting pancreatic beta cells by T cells. Despite the established role of T cells in the pathogenesis of the disease, to date, with the exception of the identification of islet-specific T effector (Teff) cells, studies have mostly failed to identify reproducible alterations in the frequency or function of T cell subsets in peripheral blood from patients with type 1 diabetes.

**Methods:**

We assessed the production of the proinflammatory cytokines IL-21, IFN-γ and IL-17 in peripheral blood mononuclear cells from 69 patients with type 1 diabetes and 61 healthy donors. In an additional cohort of 30 patients with type 1 diabetes and 32 healthy donors, we assessed the frequency of circulating T follicular helper (Tfh) cells in whole blood. IL-21 and IL-17 production was also measured in peripheral blood mononuclear cells (PBMCs) from a subset of 46 of the 62 donors immunophenotyped for Tfh.

**Results:**

We found a 21.9% (95% CI 5.8, 40.2; *p* = 3.9 × 10^−3^) higher frequency of IL-21^+^ CD45RA^−^ memory CD4^+^ Teffs in patients with type 1 diabetes (geometric mean 5.92% [95% CI 5.44, 6.44]) compared with healthy donors (geometric mean 4.88% [95% CI 4.33, 5.50]). Consistent with this finding, we found a 14.9% increase in circulating Tfh cells in the patients (95% CI 2.9, 26.9; *p* = 0.016).

**Conclusions/interpretation:**

These results indicate that increased IL-21 production is likely to be an aetiological factor in the pathogenesis of type 1 diabetes that could be considered as a potential therapeutic target.

**Electronic supplementary material:**

The online version of this article (doi:10.1007/s00125-015-3509-8) contains peer-reviewed but unedited supplementary material, which is available to authorised users.

## Introduction

Type 1 diabetes is a T cell mediated autoimmune disease characterised by exogenous insulin dependency resulting from the destruction of the insulin-producing beta cells. Pathogenesis of type 1 diabetes involves the breakdown of tolerance, autoantibody production and the activation of islet antigen-specific autoreactive CD4^+^ T cells that in turn provide help for islet antigen-specific CD8^+^ cytotoxic T cell responses [1–3]. Genetic association studies in type 1 diabetes have identified risk variants in the key genes responsible for autoimmune responses against insulin-derived peptides (HLA class II and class I genes, and the insulin gene), as well as in genes with prominent roles in CD4^+^ T cell function. Several studies have investigated proinflammatory Th1/Th17 responses in type 1 diabetes patients, reporting a Th17 bias among type 1 diabetes patients as compared with healthy individuals [4–8]. CD4^+^ T cells purified from the blood of newly diagnosed type 1 diabetes patients were skewed towards IL-17 secretion when compared with healthy controls [5, 6]; and monocytes from type 1 diabetes patients were shown to spontaneously secrete the proinflammatory cytokines IL-6 and IL-1β that induce Th17 cells [4]. There is evidence from mouse models for a role of the IL-21 pathway in type 1 diabetes [9], but aside from the established *IL2-IL21* region association with type 1 diabetes risk [10], we have only identified one published, recent study that reported increased frequencies of circulating CD4^+^ T follicular helper (Tfh) cells together with enhanced expression of IL-21 in type 1 diabetes patients [8]. This contrasts with several reports of increases in Tfh frequencies in other autoimmune diseases such as Sjogren’s syndrome, systemic lupus erythematosus (SLE), rheumatoid arthritis (RA), myasthenia gravis, autoimmune thyroid disease, juvenile dermatomyositis and multiple sclerosis [11].

In the present study, we have characterised the production of the key proinflammatory cytokines IL-21, IL-17 and IFN-γ in the peripheral T cell compartment of 69 type 1 diabetes patients and 61 healthy donors. We report here an increased production of IL-21 and, to a lesser extent, IL-17, but not IFN-γ, in memory CD4^+^ T effector (Teff) cells from type 1 diabetes patients as compared with healthy controls. Consistent with the increased production of IL-21, we also report an increased frequency of peripheral Tfh from an independent cohort of 30 long-standing type 1 diabetes patients and 32 healthy controls. Collectively, these findings suggest that Tfh cells and IL-21-mediated inflammation are involved in the pathogenesis of type 1 diabetes.

## Methods

### Participants

Adult long-standing type 1 diabetes patients (*n* = 20) and healthy controls (*n* = 21) were recruited from the Cambridge BioResource (CBR; www.cambridgebioresource.org.uk). Newly diagnosed type 1 diabetes patients (*n* = 49) and unaffected siblings (UAS) of other type 1 diabetes probands (*n* = 40) were collected from the JDRF Diabetes–Genes, Autoimmunity and Prevention (D-GAP) study (http://paediatrics.medschl.cam.ac.uk/research/clinical-trials/). Newly diagnosed patients were characterised as having been diagnosed with type 1 diabetes within less than 2 years (with one exception of 42 months) and UAS were islet autoantibody-negative, and were not related to any type 1 diabetes patient included in this study. All donors were of white ethnicity and all healthy controls were individuals without autoimmune disease (self-reported).

For the Tfh cell immunophenotyping in whole blood, an independent cohort of 30 adult long-standing type 1 diabetes patients and 32 healthy controls, were recruited from the CBR. Baseline characteristics for all participating individuals are summarised in Table 1.

**Table 1:** Baseline characteristics of study participants

Cohort	*n*	Age (years)		Male *n* (%)	Duration of disease (months)	
		Median	Range		Median	Range
Cytokine production in T1D patients and healthy controls (PBMCs)						
T1D (D-GAP)^a^	49	13	6–34	32 (65.3%)	11	2–42
T1D (CBR)^b^	20	32	22–42	8 (40.0%)	198	6–276
T1D (combined)	69	14	6–42	40 (58.0%)	22	2–276
Unaffected relatives (D-GAP)	40	13	6–31	21 (52.5%)	N/A	N/A
Healthy controls (CBR)	21	27	18–37	7 (33.3%)	N/A	N/A
Healthy controls (combined)	61	15	6–37	28 (45.9%)	N/A	N/A
T follicular helper cell immunophenotyping (whole blood)^c^						
T1D (CBR)	30	32	22–47	9 (30.0%)	204	24–240
Healthy controls (CBR)	32	32	17–52	9 (28.1%)	N/A	N/A

Baseline characteristics for the study participants stratified by the study cohorts

^a^Newly diagnosed T1D patients (duration of disease ≤3 years) enrolled in the D-GAP study

^b^Long-standing adult type 1 diabetes patients enrolled from the CBR

^c^Twenty-three of the 62 CBR donors selected for Tfh cell immunophenotyping were also assessed for the cytokine production phenotypes

N/A, not applicable; T1D, type 1 diabetes

### Ethics

All samples and information were collected with written and signed informed consent. The D-GAP study was approved by the Royal Free Hospital & Medical School Research Ethics Committee; REC (08/H0720/25). Adult long-standing type 1 diabetes patients and healthy volunteers were enrolled in the CBR. The study was approved by the local Peterborough and Fenland Research Ethics Committee (05/Q0106/20).

### Peripheral blood mononuclear cells (PBMCs) sample preparation

PBMCs were isolated by Ficoll gradient centrifugation (Lympholyte; Cederlane, Burlington, NC, USA) and cryopreserved in 10% (vol/vol) heat-inactivated human AB serum (Sigma-Aldrich, St Louis, MO, USA), as described previously [12]. Of note, type 1 diabetes patients and healthy controls were recruited contemporaneously and samples were processed and stored by the same investigators to prevent spurious findings caused by differential sample preparation.

Cryopreserved PBMCs (10 × 10^6^ per donor) were thawed in a 37°C water bath and resuspended in X-Vivo (Lonza, Basel, Switzerland) + 1% (vol/vol) heat-inactivated, filtered human AB serum. Cell viability following resuscitation was assessed in PBMCs from a subset of 40 donors using the Fixable Viability Dye eFluor 780 (eBioscience, San Diego, CA, USA) and was found to be consistently very high (median 95.6%; min 86.8%, max 98.2%) for all samples collected as part of the cohorts analysed in this study.

### Cell culture and in vitro stimulation

To reduce the effects of experimental variation and other potential covariates, PBMC samples were processed in batches of a minimum of ten samples per day. Type 1 diabetes patients and healthy controls were matched as closely as possible for age (within 5 year age bands), sex and time of sample preparation.

After thawing, PBMCs were resuspended in RPMI medium (Gibco, Paisley, UK) supplemented with 10% (vol/vol) FBS, 2 mol/l l-Glutamine and 100 μg/ml Pen-Strep and cultured (10^6^ PBMCs/well) in 24-well flat-bottom cell culture plates (BD Biosciences, San Diego, CA, USA). Cells were initially rested for 30 min at 37°C and then activated with (1) 50 ng/ml phorbol myristate acetate (PMA; Sigma-Aldrich) and 500 ng/ml ionomycin (Sigma-Aldrich) for 5 h at 37°C for IL-21 production assays; or (2) with 5 ng/ml PMA and 100 ng/ml ionomycin for 4 h at 37°C for IL-17 and IFN-γ production assays. For both stimulation conditions, 0.67 μl/ml BD GolgiStop (monensin, BD Biosciences) was added prior to cell culture. For selected samples, 10^6^ cells were cultured with medium alone and 0.67 μl/ml monensin to determine background levels of cytokine production in unstimulated cells. For a subset of 39 samples assessed in the later two batches, IFN-γ- and IL-17-producing cells were also assessed in parallel using a higher stimulation condition (10 ng/ml PMA and 500 ng/ml ionomycin).

**Fig. 1:**
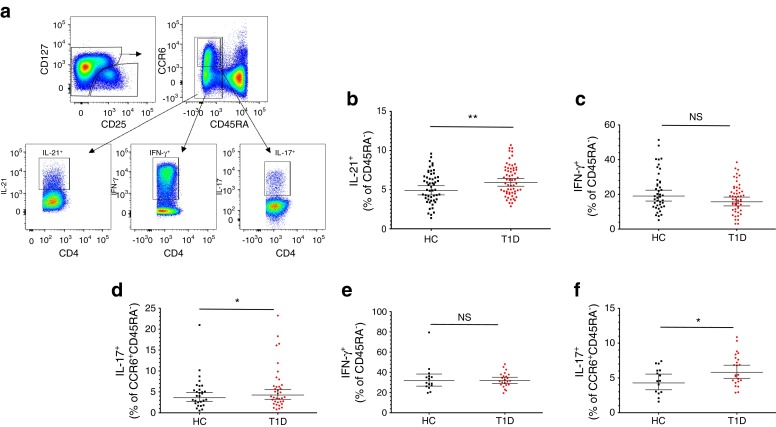
IL-21 production is increased in type 1 diabetes patients. (**a**) Gating strategy for the delineation of IL-21^+^ CD45RA^−^, IFN-γ^+^ CD45RA^−^ and IL-17^+^ CD45RA^−^ CCR6^+^ CD4^+^ memory effector T cell subsets. (**b**) Scatter plot depicts the distribution (geometric mean ± 95% CI) of IL-21^+^ cells among memory CD45RA^−^ CD4^+^ T cells. The frequency of IL-21^+^ cells was compared between 61 type 1 diabetes patients and 53 healthy donors (*p* = 3.9 × 10^−3^). (**c**, **d**) Scatter plots depict the frequency of IFN-γ^+^ (**c**) and IL-17^+^ (**d**) cells in a sample of 62 type 1 diabetes patients and 54 healthy donors following in vitro stimulation with 100 ng/ml PMA and ionomycin. (**e**, **f**) Frequency of IFN-γ^+^ (**e**) and IL-17^+^ (**f**) cells was also assessed in a subset of 24 type 1 diabetes patients and 15 healthy controls following in vitro stimulation with a higher concentration (500 ng/ml) of ionomycin. The *p* values were calculated by linear regression of the log-transformed data, including batch as a covariate. Horizontal bars represent the geometric mean (±95% CI) obtained from the transformation of the log-transformed data: (*Y* = exp[*Y*]) of each group. Additional data from the statistical analysis are provided in Table 3. HC, healthy control; T1D, type 1 diabetic patient. *p < 0.05, **p < 0.01

We included one unstimulated sample in each batch, which was used to assess staining of IL-21, IL-17 and IFN-γ, and set the gate for cytokine-positive cells. As expected in these short-term cultures, we observed little production of cytokines in unstimulated sells (Electronic Supplementary Material [ESM] Fig. 1).

The concentrations of PMA and ionomycin used for in vitro stimulation in this study were optimised to ensure optimal cytokine production and minimal cell death. Even at the highest activation condition used in this study (50 ng/ml PMA and 500 ng/ml ionomycin for 5 h), cell viability was found to be sufficient for analysis (median 80.8% live cells; min 37.2%, max 94.3%) following cell culture and in vitro stimulation.

### Intracellular immunostaining

After activation, PBMCs were harvested, and stained with Fixable Viability Dye eFluor 780 for 20 min at 4°C. Cells were then stained with fluorochrome-conjugated antibodies against surface receptors (see Table 2) for 1 h at 4°C. Fixation and permeabilisation was performed using forkhead box protein 3 (FOXP3) Fix/Perm Buffer Set (BioLegend, San Diego, CA, USA) and cells were then stained with the respective intracellular antibodies or isotype control antibody for 1 h at 4°C (see Table 2).

**Table 2:** Antibodies and immunostaining panels used for flow cytometry

Immunostaining panel	Antibody	Fluorochrome	Clone	Manufacturer
IL-21	CD4	AF700	RPA-T4	BioLegend
	CD25^a^	APC	M-A251 + 2A3	BD Biosciences
	CD127	PE-Cy7	eBioRDR5	eBioscience
	CD45RA	AF488	HI100	BioLegend
	IL-21 (IC)	PE	3A3-N2 (MOPC-21)	BioLegend
	FOXP3	PB	259D	BioLegend
	Viability dye	eFluor780	–	eBioscience
IFN-γ/IL-17	CD4	AF700	RPA-T4	BioLegend
	CD25^a^	APC	M-A251 + 2A3	BD Biosciences
	CD127	PE-Cy7	eBioRDR5	eBioscience
	CD45RA	BV785	HI100	BioLegend
	CCR6	BV605	G034E3	BioLegend
	FOXP3	PB	259D	BioLegend
	HELIOS	FITC	22F6	BioLegend
	IFN-γ	PE	B27	BD Biosciences
	IL-17	PerCP Cy5.5	BL168	BioLegend
	Viability dye	eFluor780	–	eBioscience
Tfh cell immuno-phenotyping (whole blood)	PD-1	PE	MIH4	BD Biosciences
	CD4	AF700	RPA-T4	BioLegend
	CD45RA	PE-Cy7	HI100	BioLegend
	CD25^a^	APC	M-A251 + 2A3	BD Biosciences
	CCR6	AF488	G034E3	BioLegend
	CD3	eFluor780	SK7	eBioscience

Anti-human monoclonal antibodies used for the T-cell immunophenotyping

^a^Two clones of anti-CD25 that bind to different epitopes were used simultaneously to enhance CD25 staining

AF, Alexa Fluor; APC, allophycocyanin; BV, Brilliant Violet; Cy, cyanine; IC, isotype control; PB, Pacific Blue; PE, phycoerythrin; PerCP, peridinin-chlorophyll proteins

### Tfh cell surface immunostainings

For the Tfh cell immunophenotyping, up to two adult long-standing type 1 diabetes patients and at least one age- and sex-matched healthy donor were recruited on the same day from the CBR. Whole blood was collected by venipuncture and stained within 2 h of collection with fluorochrome-conjugated monoclonal antibodies (Table 2) for 40 min at 4°C. Erythrocytes were then lysed and the white blood cells fixed for 10 min at room temperature using BD FACS lysing solution (BD Biosciences).

### Flow cytometry

Immunostained samples were acquired using a BD Fortessa (BD Biosciences) flow cytometer with FACSDiva software (BD Biosciences). Flow cytometry data were exported in format 3.0 and analysed using FlowJo (Tree Star, Ashland, OR, USA). Compensation controls were generated using CompBeads (BD Biosciences) compensation beads. Cyto-Cal calibration beads (Thermo Fisher Scientific, Waltham, MA, USA) were used to assess instrument stability. Dead-cell exclusion based on the Fixable Viability Dye was performed for the intracellular immunostainings.

### Statistical analyses

Statistical analyses were performed using Prism software (GraphPad, La Jolla, CA, USA) and Stata (www.stata.com). Association of the assessed cytokine production phenotypes with type 1 diabetes was calculated by linear regression including batch as a covariate. The effects of age, sex and time of collection on the association with type 1 diabetes were dealt by the experimental design used in this study and, therefore, not included as additional covariates. Given that most cytokine production phenotypes showed moderate to strong right skew that violated the assumption of normality, the phenotypes were log-transformed (natural log) before statistical testing. Three donors (two type 1 diabetes patients and one healthy donor) were found to carry a known polymorphism in *PTPRC* (*CD45*), which reduces production of the alternatively spliced mRNA encoding the CD45RO isoform in memory cells thereby increasing the expression of CD45RA on this subset. Since the delineation of most assessed phenotypes depended on the surface expression of CD45RA, these three donors were excluded from the analysis.

The frequency of circulating Tfh cells between type 1 diabetes patients and healthy controls was compared using an unpaired two-tailed Student’s *t* test.

## Results

### IL-21 production is increased in CD4^+^ memory effector T cells from type 1 diabetes patients

We measured the production of three major proinflammatory cytokines, IL-21 (*n* = 114), IL-17 (*n* = 66) and IFN-γ (*n* = 116), by polychromatic flow cytometry in type 1 diabetes patients and healthy controls, matched as closely as possible for age, sex and time of sample preparation (Table 1). All phenotypes were assessed in thawed, cryopreserved PBMCs following in vitro activation with PMA and ionomycin. Consistent with the age-matching of cases and controls in this study, we observed no significant difference in the overall frequency of memory (CD45RA^−^) CD4^+^ Teff cells in type 1 diabetes cases compared with healthy controls (*p* = 0.15; ESM Fig. 2). To avoid increased variability caused by CD4^+^ T cell subset heterogeneity and intra-individual variation in the composition of the immune subsets, we have normalised our data on the memory CD45RA^−^ CD4^+^ Teff population for IL-21 and IFN-γ and the CCR6^+^ subset of this same memory population for IL-17, a chemokine receptor expressed ubiquitously on Th17 cells [13] (Fig. 1a). We found that the frequency of IL-21^+^ cells within the memory CD45RA^−^ Teff cell population was increased in type 1 diabetes patients (geometric mean 5.92% [95% CI 5.44, 6.44]) compared with healthy donors (geometric mean 4.88% [95% CI 4.33, 5.50], *p* = 3.9 × 10^−3^; Fig. 1b and Table 3). This result suggests that a larger proportion of CD4^+^ Teff cells from type 1 diabetes patients as compared with healthy donors have previously secreted IL-21 in response to stimulation within various tissues, including the islets and pancreatic lymph nodes. The frequency of IL-21^+^ CD4^+^ Teffs was not associated with the duration of the disease, ranging from 2 months to 23 years in our patient population (*p =* 0.78*)*.

**Table 3:** Association analysis of the peripheral CD4^+^ T-cell compartment with T1D

Phenotype (gating strategy)	*n*	Healthy donors		Type 1 diabetes patients		% change (95% CI)^a^	*p* value
		*n*	Mean frequency (95% CI)	*n*	Mean frequency (95% CI)		
Cytokine production^b^							
IL-21^+^ memory T cells (% of CD4^+^) (CD4^+^ CD45RA^−^ IL21^−^)	114	53	4.88% (4.33, 5.50)	61	5.92% (5.44, 6.44)	21.9% (5.8, 40.2)	3.9 × 10^−3^
IFN-γ^+^ memory T cells (% of CD4^+^) (CD4^+^ CD45RA^−^ IFN-γ^+^)	116	54	20.12% (17.52, 23.11)	62	16.93% (14.67, 19.52)	−12.1% (−24.0, 1.7)	0.07
IL-17^+^ CCR6^+^ memory T cells (% of CD4^+^) (CD4^+^ CD45RA^−^ CCR6^+^ IL17^+^)	66	29	3.61% (2.68, 4.86)	37	4.26% (3.25, 5.58)	28.7% (−0.7, 65.9)	0.04
CD4^+^ T follicular helper cell (Tfh) frequency^c^							
Tfh (% of CD4^+^ CD45RA^−^ CXCR5^+^) (PD-1^+^ CCR6^−^)	62	32	26.02% (23.86, 28.15)	30	29.89% (27.53, 32.25)	14.9% (2.9, 26.9)	0.016

The *p* values for the cytokine production phenotypes were calculated by linear regression, including batch as a covariate, comparing the mean frequency of the assessed cytokine production phenotypes in type 1 diabetes patients and healthy donors matched as closely as possible for age, sex and time of sample preparation

^a^Percent change and 95% CI of the respective T cell phenotype in type 1 diabetes patients relative to healthy donors

^b^Statistical tests for the cytokine production phenotypes were performed on log-transformed data because some phenotypes showed a strong right skew. Mean frequencies represent the geometric means obtained from the transformation (*Y* = exp[*Y*]) of the log-transformed data (natural log)

^c^The *p* value for the Tfh cell immunophenotyping was calculated using an unpaired two-tailed Student’s *t* test

### Production of IL-17 and IFN-γ in CD4^+^ memory cells of type 1 diabetes patients and healthy donors

In contrast to IL-21, there was no convincing support for a differential frequency of IFN-γ-producing cells within the CD45RA^−^ CD4^+^ T cell subset (*p* = 0.07) in type 1 diabetes patients as compared with controls (Fig. 1c and Table 3). To investigate if this lack of association with type 1 diabetes was also observed under stronger activation conditions, we also measured the production of IFN-γ in a subset of 38 donors in whom cytokine production was assessed using more potent PBMC stimulation conditions (500 ng/ml ionomycin, instead of 100 ng/ml). Similarly, we found no difference for the production of IFN-γ by CD45RA^−^ CD4^+^ T cells between type 1 diabetes patients and healthy controls under these experimental conditions (*p* = 0.90; Fig. 1e). We did observe an increase in the frequency of IL-17^+^ cells within the subpopulation of CD45RA^−^ CCR6^+^ T cells in type 1 diabetes patients (geometric mean 4.26% [95% CI 3.25, 5.58]) compared with healthy donors (geometric mean 3.61% [95% CI 2.68, 4.86], *p* = 0.04; Fig. 1d and Table 3), which was also replicated (*p* = 0.03; Fig. 1f) in the subset of 38 donors activated using different PBMC stimulation conditions (500 ng/ml ionomycin).

**Fig. 2:**
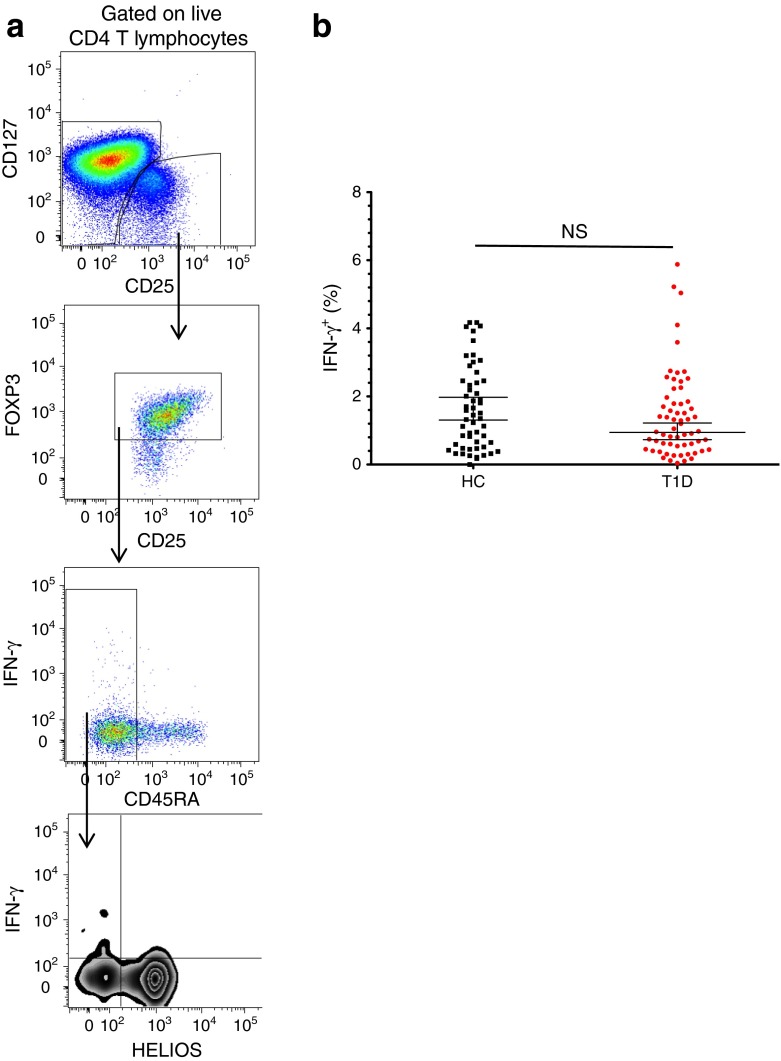
Frequency of IFN-γ^+^ HELIOS^−^ CD45RA^−^ FOXP3^+^ CD4^+^ Tregs is not altered in type 1 diabetes patients. (**a**) Gating strategy for the HELIOS^−^ CD45RA^−^ FOXP3^+^ CD4^+^ Treg subset. FACS gating plots depict data from one illustrative donor. (**b**) Scatter plot depicts the distribution (geometric mean ± 95% CI) of IFN-γ^+^ cells in the HELIOS^−^CD45RA^−^ FOXP3^+^ CD4^+^ Treg subset. The frequency of IFN-γ^+^ cells was compared between type 1 diabetes patients (*n* = 62) and healthy donors (*n* = 54; *p* = 0.79). The *p* values were calculated by linear regression of the log-transformed data, including batch as a covariate. HC, healthy control; T1D, type 1 diabetic patient

In addition to the production of IFN-γ by CD45RA^−^ CD4^+^ T cells, we also investigated its production in the subset of CD127^−^ CD25^+^ FOXP3^+^ regulatory T cells (Tregs), according to the expression of the transcription factor HELIOS (Fig. 2a). Consistent with their regulatory phenotype, we found that the HELIOS^+^ FOXP3^+^ Tregs did not produce the proinflammatory cytokines IFN-γ and IL-17, after in vitro stimulation. In contrast, a small proportion of the HELIOS^−^ FOXP3^+^ fraction was able to produce IFN-γ, which was pronounced in the memory CD45RA^−^ subset. Nevertheless, we did not observe a difference in IFN-γ production by HELIOS^−^ CD45RA^−^ FOXP3^+^ Tregs between type 1 diabetes patients and healthy controls (*p* = 0.79; Fig. 2b).

### Increased IL-21 production is associated with an increased frequency of circulating Tfh cells within the memory cell population in type 1 diabetes patients

**Fig. 3:**
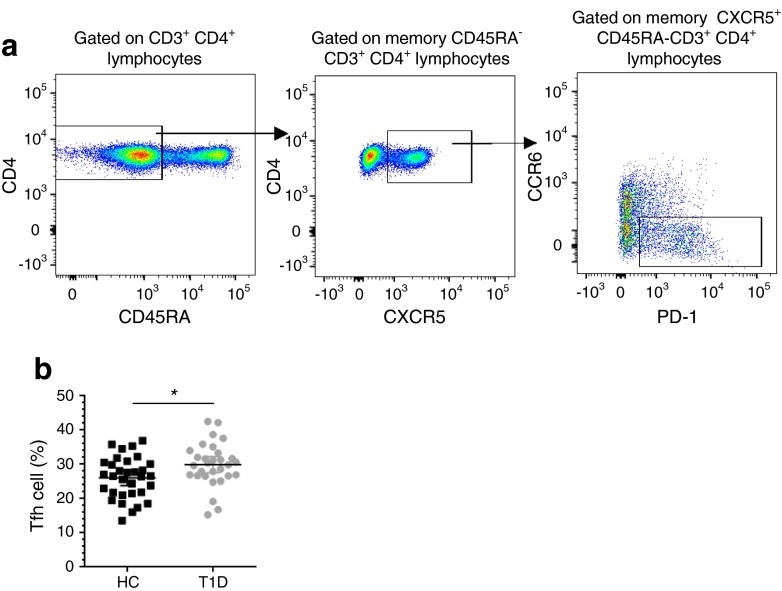
Increased frequency of Tfh cells within the CXCR5^+^ CD4^+^ memory cell subset of type 1 diabetes patients as compared with healthy controls. (**a**) Gating strategy for the delineation the Tfh memory effector T cell subset defined as the frequency of PD-1^+^ CCR6^−^ cells out of CD45RA^−^ CXCR5^+^ CD4^+^ T cells. (**b**) Scatter plot depicts the distribution (geometric mean ± 95% CI) of Tfh cells (defined as % PD-1^+^ of CCR6^−^ CXCR5^+^) in a cohort of type 1 diabetes patients (*n* = 30) and healthy controls (*n* = 32; *p* = 0.016). Frequency of circulating Tfh cells was assessed using fresh whole blood from type 1 diabetes patients and healthy donors enrolled from the CBR, matched for sex and 5 year age bands. The *p* value was calculated using an unpaired two-tailed *t* test. HC, healthy control; T1D, type 1 diabetic patient. *p < 0.05

Since IL-21 production is characteristic of Tfh cells, we examined the frequency of this subset in whole blood samples from an independent cohort of 30 type 1 diabetes patients and 32 healthy donors. Consistent with the increase in the frequency of IL-21-producing cells within the memory CD4^+^ T cell subset, we observed an increased frequency of Tfh cells, known to produce IL-21 and defined as PD-1^+^ CCR6^−^ cells, from CD45RA^−^ CXCR5^+^ CD4^+^ T cells in type 1 diabetes patients (mean 29.89% [95% CI 27.53, 32.25]) compared with age- and sex-matched healthy controls (mean 26.02% [95% CI 23.86, 28.15], *p* = 0.016; Fig. 3 and Table 3), suggesting that IL-21 production by Tfh cells may be an aetiological factor in a subset of type 1 diabetes patients characterised by increased Tfh cell frequency in the peripheral circulation.

To investigate the potential association between frequency of circulating Tfh cells and IL-21^+^ memory T cells, we measured IL-21 production in cryopreserved PBMCs from 46 (24 type 1 diabetes patients and 22 healthy controls) of the 62 individuals who had been assessed for Tfh frequency. Importantly, to avoid the effect of day-to-day variation associated with cytokine production assays, we measured the frequency of IL-21^+^ memory T cells in a single experimental batch. In this sample, we found that frequency of Tfh cells was associated with the frequency of IL-21^+^ cells (*r* ^2^ = 0.174, *p* = 0.004; ESM Fig. 3), suggesting that although not the only source of IL-21, Tfh cells contribute to the production of IL-21 observed in our study.

In addition to investigating the potential correlation between the frequency of Tfh and IL-21^+^ memory T cells, we investigated whether differential expression of IL-21 in type 1 diabetes patients could be caused by an altered T central memory (Tcm) cell compartment, a subset that has been recently found to have a higher proportion of IL-21-producing cells compared with the T effector memory (Tem) subset [14]. Owing to compromised CD62L and CCR7 staining in cryopreserved PBMCs, we used CD27 as a marker of Tcm cells [15] (ESM Fig. 4a). In these 46 donors, we found no evidence for an altered frequency of CD27^+^ Tcm cells within the CD45RA^−^ Teff compartment (*p* = 0.717; ESM Fig. 4b).

We also found that the frequency of IL-21^+^ cells among the Tcm subset was still significantly increased in type 1 diabetes patients (geometric mean 5.33% [95% CI 4.75, 5.98] and 4.21% [95% CI 3.81, 4.66], respectively, *p* = 2.8 × 10^−3^; ESM Fig. 4c). Furthermore, under our stimulation conditions both the Tcm and Tem subsets, defined based on the expression of CD27 on our sample of cryopreserved PBMCs, were able to produce IL-21 (geometric mean 5.0% and 8.2%, respectively). Taken together, these data indicate that our observed increase in the frequency of IL-21^+^-producing cells within the CD45RA^−^ memory Teff cell subset is not caused by an altered composition of the CD45RA^−^ memory Teff compartment in type 1 diabetes patients.

## Discussion

Systemic alterations in the peripheral immune system of autoimmune disease patients have proved challenging to identify, particularly in organ-specific autoimmune diseases such as type 1 diabetes, in which onset of the first disease symptoms, namely autoantibodies, can precede clinical diagnosis by many years. In the present study, following in vitro stimulation, we detected an increased proportion of IL-21-producing cells within the memory CD4^+^ Teff population in type 1 diabetes patients. IL-21 is a well-established Tfh cytokine that plays a major role in the generation of germinal centre reactions and antibody production [16], and Tfh cell differentiation has been previously shown to be negatively regulated by IL-2 [17–19]. As a consequence, the demonstration of increased IL-21 production in type 1 diabetes patients supports the findings from genetic studies of type 1 diabetes implicating a dysfunction of the IL-2 signalling pathway in the pathogenesis of the disease [20–22]. It will be important to determine if the variants mapping to the *IL2-IL21* region that influence susceptibility to type 1 diabetes and other autoimmune diseases [10, 23–25] directly influence IL-2 or IL-21 production in a cell-intrinsic manner. In addition to promoting B cell responses, IL-21 has also been shown to be an important factor for the differentiation of the Th17 lineage [6, 26, 27]; therefore, the increase in IL-17-producing cells in type 1 diabetes patients as compared with healthy controls observed in the current study, as well as in previous studies [4–6], could be a consequence of increased IL-21 production. Results from a recent study emphasised the pleiotropic nature of IL-21: IL-21 promoted Th17 lineage differentiation and IL-10 production while inhibiting Th1 differentiation as well as the generation of potentially pathogenic Th1/17 effector cells [14]. How these influences on T cell differentiation and effector function by IL-21 interact in an in vivo setting and contribute to autoimmune disease pathogenesis, especially balanced against the ability of IL-21 to promote B cell differentiation and antibody production, requires further investigation.

A limitation of the current study is that we are not able to provide a functional mechanism to support the suggested involvement of IL-21 in the aetiology of the disease. This question will need to be addressed in future mechanistic studies designed at characterising circulating Tfh cells in human type 1 diabetes patients in vivo, and exploring the clinical outcomes associated with the subset of type 1 diabetes patients with increased Tfh cell frequency and increased IL-21 levels. An intriguing possibility is that the effect of inherited genetic risk variants leading to deficient regulation of IL-2 signalling are manifested in the increased production of IL-21 by Tfh cells. Previously, we showed that variants in *IL2RA* that predispose to type 1 diabetes reduce the level of IL-2RA/CD25 on Tregs and memory Teff cells [20, 22], thereby potentially increasing the amount of homeostatic IL-2 production required for Treg survival and function, and limiting Tfh differentiation.

Overall, our findings underscore an inherent bias towards a proinflammatory response in type 1 diabetes patients and potentially reflect alterations of the immune system observed in the autoimmune microenvironment, which are critically dependent on IL-21 signalling [28]. In agreement with this hypothesis, we show that the frequency of Tfh cells is increased on average by 14.9% in type 1 diabetes patients compared with controls, consistent with a recent study [8]. The relevance of this Tfh phenotype to type 1 diabetes pathogenesis is strongly supported by previous reports showing increased levels of Tfh cells in seven other autoimmune diseases [11]. Their increased prevalence in the circulation in other diseases also reduces the possibility that this Tfh phenotype, as well as the increased IL-21 production in type 1 diabetes patients shown here and previously reported [8], are a consequence of insulin treatment, a potential confounding factor in type 1 diabetes case–control studies. One of the strongest type 1 diabetes associated loci, *PTPN22,* may also be implicated in Tfh cell responses and IL-21 production leading to an increase in B cell numbers and antibody production in *Ptpn22* knockout mice [29]. It is plausible that the increased frequency of circulating Tfh cells is directly responsible for the increased IL-21 production observed in type 1 diabetes patients. Of the 62 donors recalled for Tfh immunophenotyping, we were able to measure the frequency of IL-21^+^ CD45RA^−^ T cells in 46 of them, and found a modest correlation between the frequencies of circulating Tfh and IL-21 CD45RA^−^ memory T cells. These data suggest that, although Tfh cells are likely an important source of this cytokine, there are also additional cell types that also contribute to IL-21 production, as previously described [11].

In contrast to IL-21 and, to a lesser extent IL-17, we found no evidence for the differential expression of the proinflammatory cytokine IFN-γ in type 1 diabetes patients. This lack of association was observed not only among CD45RA^−^ CD4^+^ Teff cells, but also among a subset of HELIOS^−^ CD45RA^−^ FOXP3^+^ CD4^+^ T cells. The ability of HELIOS^−^ FOXP3^+^ CD4^+^ T cells to produce IFN-γ is consistent with similar observations by McClymont et al who reported that HELIOS^−^ FOXP3^+^ CD4^+^ Tregs produced increased levels of IFN-γ in type 1 diabetes cases [7].

In summary, we have identified an imbalance in IL-21 production in type 1 diabetes patients, which is accompanied by an increased frequency of circulating Tfh cells. Although this imbalance in the periphery is only manifested in a 21.9% increase in the frequency of IL-21^+^ effector memory CD4^+^ T cells in patients, it is likely the reflection of a much larger effect on secondary lymphoid organs, particularly at disease diagnosis. In mice, IL-21 inhibits T cell IL-2 production and impairs Treg homeostasis [30]. Taken together with findings in other autoimmune diseases [11], there is genetic and immunological rationale for the initiation of clinical trials to investigate the safety and efficacy of anti-IL-21 therapy in RA patients (www.clinicaltrials.gov - NCT01565408 and NCT01647451) and in other diseases, including type 1 diabetes. Interestingly, B cell depletion with a course of anti-CD20 therapy (rituximab) decreased circulating IL-21 levels and the frequency of Tfh cells in peripheral blood of type 1 diabetes patients, and preserved beta cell function in ten out of 20 patients [8]. The authors proposed that the observed effects of anti-CD20 reflect close interactions between Tfh and B cells in germinal centres. For example, in mice, IL-21 is a potent inducer of the co-stimulatory molecule CD86 on B cells thereby promoting antigen presentation [31]. In light of previous evidence of some clinical efficacy of anti-CD20 therapy in type 1 diabetes patients [32], it is possible that pre-selected patients stratified on the basis of increased levels of IL-21, Tfh cells or with multiple circulating autoantibodies and IFN-γ production [33] could benefit to a greater extent from clinical interventions, such as B cell depletion, IL-2 replacement or anti-IL-21 therapy.

## Electronic supplementary material

Below is the link to the electronic supplementary material.

ESM Fig. 1 (PDF 231 kb)

ESM Fig. 2 (PDF 170 kb)

ESM Fig. 3 (PDF 81 kb)

ESM Fig. 4 (PDF 171 kb)

## Abbreviations

CBR: Cambridge BioResource
D-GAP: Diabetes–Genes Autoimmunity and Prevention
FOXP3: Forkhead box protein 3
PBMC: Peripheral blood mononuclear cell
PMA: Phorbol myristate acetate
RA: Rheumatoid arthritis
Tfh: T follicular helper
Teff: T effector
Tcm: T central memory
Tem: T effector memory
Tregs: Regulatory T cells
UAS: Unaffected sibling
