# Supplementary material for: IL-21 production by CD4+ effector T cells and frequency of circulating follicular helper T cells are increased in type 1 diabetes patients
Source: Diabetologia. 2015 Feb 6;58(4):781–90. doi: 10.1007/s00125-015-3509-8 (PMC4351433; doi:10.1007/s00125-015-3509-8)
Supplement: Supplementary file 1 — (PDF 231 kb) [file 125_2015_3509_MOESM1_ESM.pdf]

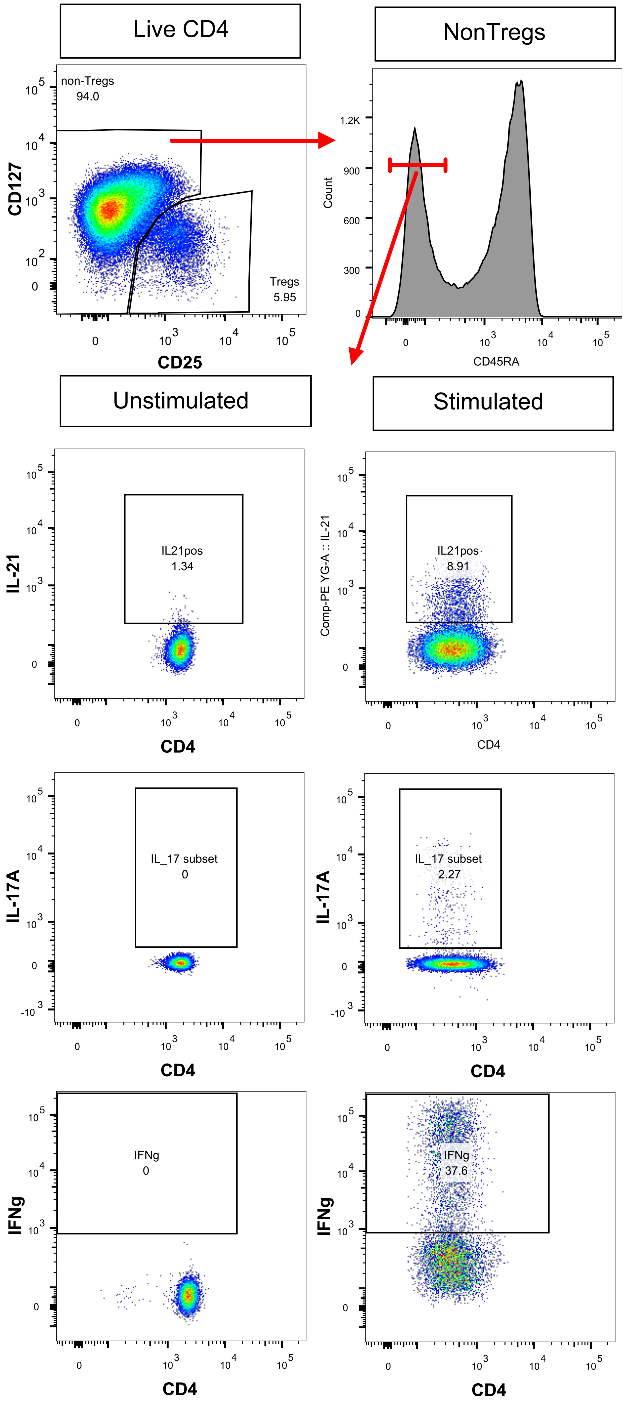

Live CD4
NonTregs
non-Tregs
94.0
Tregs
5.95
CD127
CD25
Count
CD45RA
Unstimulated
Stimulated
IL21pos
1.34
IL21pos
8.91
IL-21
Comp-PE YG-A :: IL-21
CD4
IL_17 subset
0
IL_17 subset
2.27
IL-17A
IFNg
0
IFNg
37.6

ESM Figure 1. Cytokine stainings in unstimulated cells. Plots depict the gating strategy used in this study to delineate the $CD4^+$ $CD45RA^-$ Teff compartment where cytokine expression levels were measured. Background staining for the three assessed cytokines, IL-21 (top panels), IL-17 (middle panels) and IFN-γ (bottom panels) were determined by comparing the staining levels in both unstimulated cells and in cells stimulated with phorbol-12-myristate-13-acetate (PMA) and ionomycin.
