# Supplementary material for: IL-21 production by CD4+ effector T cells and frequency of circulating follicular helper T cells are increased in type 1 diabetes patients
Source: Diabetologia. 2015 Feb 6;58(4):781–90. doi: 10.1007/s00125-015-3509-8 (PMC4351433; doi:10.1007/s00125-015-3509-8)
Supplement: Supplementary file 2 — (PDF 170 kb) [file 125_2015_3509_MOESM2_ESM.pdf]

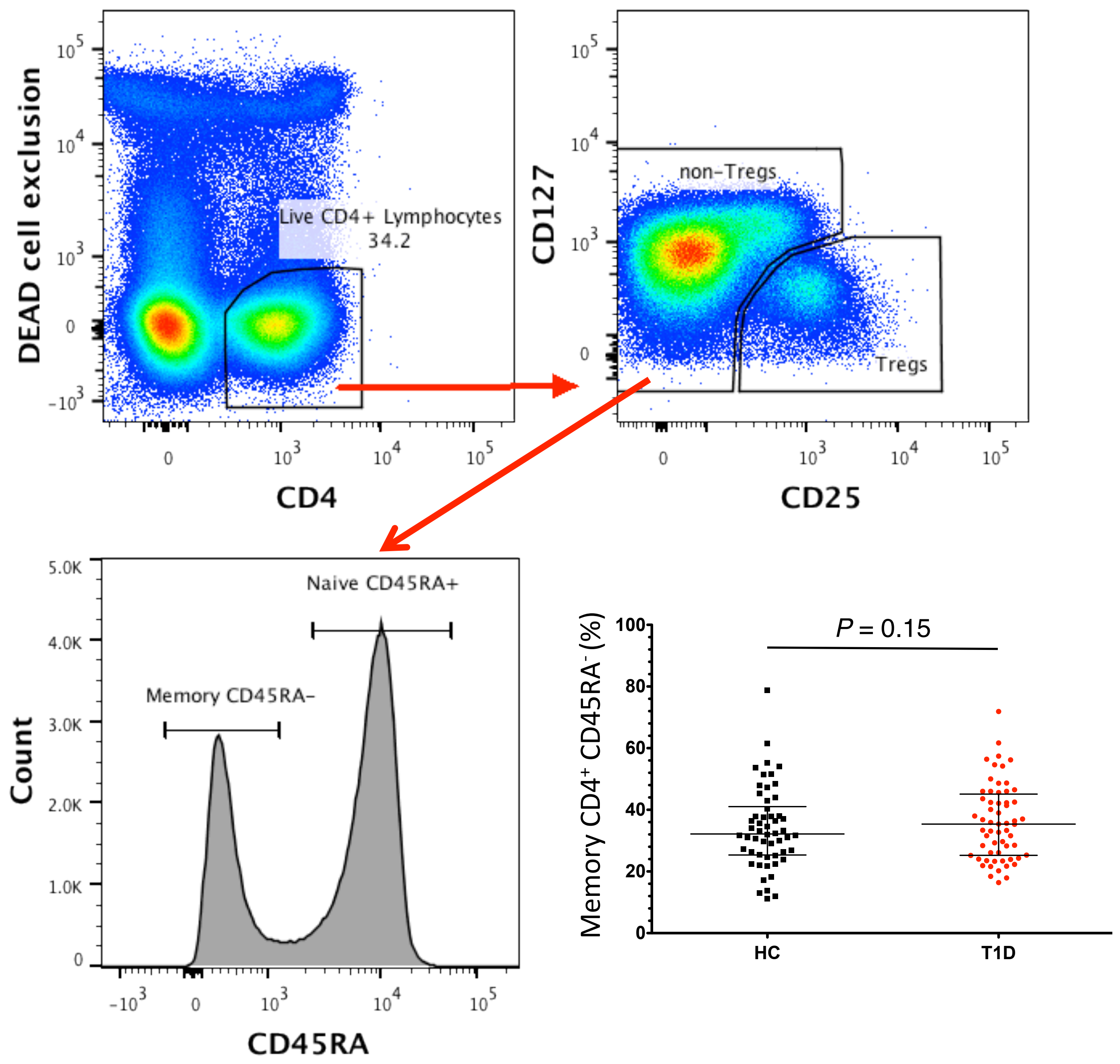
DEAD cell exclusion
CD4
Live CD4+ Lymphocytes
34.2
CD127
CD25
non-Tregs
Tregs
Count
CD45RA
Memory CD45RA–
Naive CD45RA+
Memory CD4+ CD45RA- (%)
P = 0.15
HC
T1D

ESM Figure 2. CD45RA$^-$ compartment is not affected in T1D patients. Gating strategy for the delineation of the CD45RA$^-$ memory T-cell compartment in this study. Histogram depicts the distribution of CD45RA expression in one illustrative donor and the respective frequency of memory CD45RA$^-$ and naïve CD45RA$^+$ cells. Frequency of the CD45RA$^-$ memory subset (geometric mean +/- 95% CI) was compared in T1D patients (N = 62) and healthy donors (N = 54; P = 0.15). P value was calculated by linear regression of the log-transformed data, including batch as a covariate. All data were derived from PBMCs following in vitro stimulation with phorbol-12-myristate-13-acetate (PMA) and ionomycin. The initial CD4$^+$ gate (CD4 versus dead cell exclusion dye) was derived from a lymphocyte gate (defined on forward and side scatter) followed by single-cell discrimination. FACS gating plots are representative of one illustrative individual. HC, healthy control; T1D, type 1 diabetic patient.
