# Supplementary material for: IL-21 production by CD4+ effector T cells and frequency of circulating follicular helper T cells are increased in type 1 diabetes patients
Source: Diabetologia. 2015 Feb 6;58(4):781–90. doi: 10.1007/s00125-015-3509-8 (PMC4351433; doi:10.1007/s00125-015-3509-8)
Supplement: Supplementary file 3 — (PDF 81 kb) [file 125_2015_3509_MOESM3_ESM.pdf]

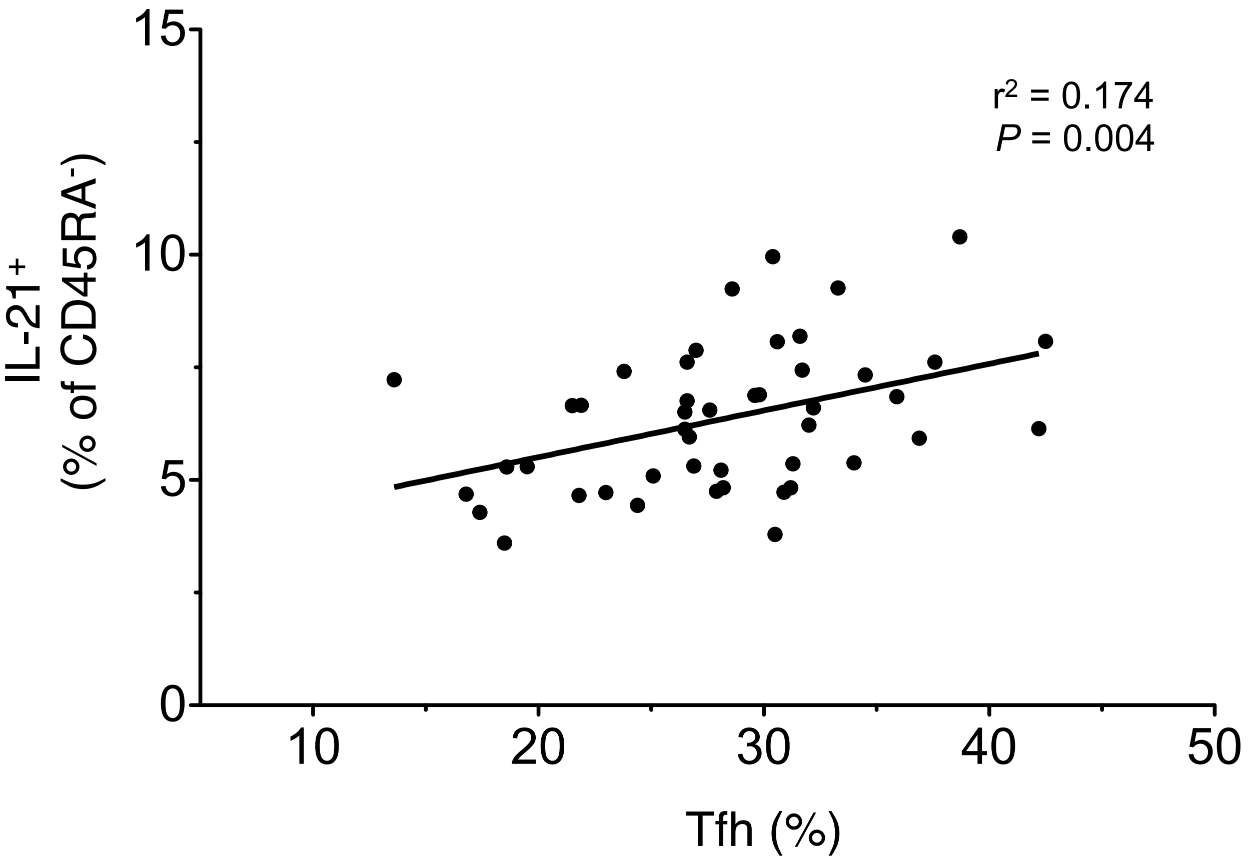

IL-21+
(% of CD45RA-)
15
10
5
0
10
20
30
40
50
Tfh (%)
r2 = 0.174
P = 0.004

ESM Figure 3. Frequency of IL-21^{+} memory T cells is correlated with the frequency of circulating Tfh cells. Frequency of IL-21^{+} memory T cells was measured in cryopreserved PBMCs from 46 donors collected at the same visit where the frequency of circulating Tfh cells (defined as the frequency of PD-1^{+}CCR6^{-} cells out of CXCR5^{+} CD45RA^{-} CD4^{+} T cells) was assessed in whole blood. IL-21^{+} frequency was measured by intracellular flow cytometry following in vitro stimulation with phorbol-12-myristate-13-acetate (PMA) and ionomycin. All samples were processed in a single batch. *P* value was calculated using a linear regression analysis.
