# Supplementary material for: IL-21 production by CD4+ effector T cells and frequency of circulating follicular helper T cells are increased in type 1 diabetes patients
Source: Diabetologia. 2015 Feb 6;58(4):781–90. doi: 10.1007/s00125-015-3509-8 (PMC4351433; doi:10.1007/s00125-015-3509-8)
Supplement: Supplementary file 4 — (PDF 171 kb) [file 125_2015_3509_MOESM4_ESM.pdf]

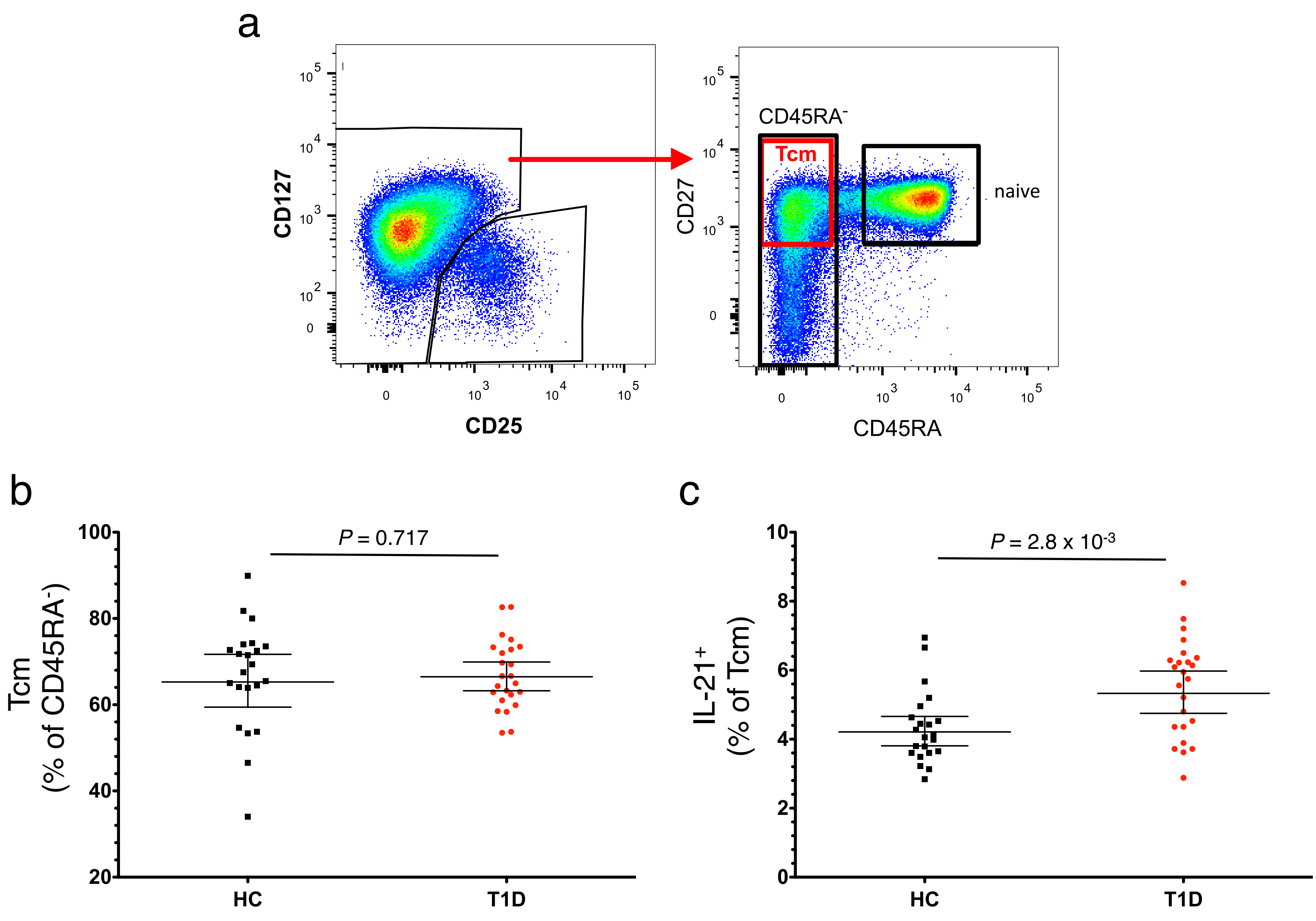
a
CD127
CD25
CD27
CD45RA
CD45RA-
Tcm
naive
b
Tcm
(% of CD45RA-)
P = 0.717
HC
T1D
c
IL-21+
(% of Tcm)
P = 2.8 x 10-3
HC
T1D

ESM Figure 4. Frequency of the CD45RA$^-$CD27$^+$ central memory T cell (Tcm) compartment is not altered in T1D patients. (**a)** Gating strategy for the delineation of the CD27$^+$ Tcm subset out of the total CD45RA$^-$ memory T cell compartment in cryopreserved PBMCs following in vitro stimulation with PMA and ionomycin. (**b**) Frequency of the Tcm memory subset (geometric mean +/- 95% CI) was compared in T1D patients (N = 24) and healthy donors (N = 22). (**c**) Frequency of IL-21$^+$ Tcm cells (geometric mean +/- 95% CI) was compared in T1D patients (N = 24) and healthy donors (N = 22). *P* values were calculated by linear regression of the log-transformed data. HC, healthy control; T1D, type 1 diabetic patient.
